# Supplementary material for: Associations between neighborhood social capital, oral health risk factors, and tooth decay among Medicaid-enrolled adolescents: A hypothesis-generating preliminary study
Source: PLoS One. 2025 Aug 13;20(8):e0329830. doi: 10.1371/journal.pone.0329830 (PMC12348974; doi:10.1371/journal.pone.0329830)
Supplement: S1 File — S1 Table. Survey items used to construct each neighborhood-level social capital measure. S2 Table. Mediating effects of worrying about food money between neighborhood social capital and tooth decay with interaction between neighborhood social capital and worrying about food money for Oregon Medicaid-enrolled adolescents ages 12–18 years who participated in a neighborhood oral health study in 2015–2016 (n = 299). S3 Table. Mediating effects of worrying about food money between neighborhood social capital and DMFS for Oregon Medicaid-enrolled adolescents ages 12–18 years who participated in a neighborhood oral health study in 2015–2016 (n = 299). (DOCX) [file pone.0329830.s001.docx]

**SUPPORTING INFORMATION**

**Table S1. Survey items used to construct each neighborhood-level social capital measure.**

| Neighborhood social capital forms | Survey items |
| --- | --- |
| Social support | (1) People around here are willing to help their neighbors  (2) How often do you and people in your neighborhood do favors for each other?  (3) People show support for their neighbors when bad things happen to them  (4) If I were sick, I could count on my neighbors to shop for groceries for me  (5) If I were sick, I could count on my neighbors to drive me to the doctor  (6) If children were skipping school, how likely is it that neighbors would do something about it? |
| Social leverage | (1) How often do you and other people in the neighborhood ask each other advice about: Child rearing?  (2) How often do you and other people in the neighborhood ask each other advice about: Job openings and/or job training program?  (3) How often do you and other people in the neighborhood ask each other advice about: Financial matters?  (4) How often do you and other people in the neighborhood ask each other advice about: Health insurance matters  (5) How often do you and other people in the neighborhood ask each other advice about: Social programs (e.g. Medicaid, Medicare, unemployment/disability assistance, social security)? |
| Informal social control | (1) If children were skipping school, how likely is it that neighbors would do something about it?  (2) If children were spray painting graffiti on a local building, how likely is it that your neighbors would do something about it?  (3) If a child was showing disrespect to an adult, how likely is it that people in your neighborhood would scold that child? |
| Organization participation | (1) Participated in a neighborhood or block organization meeting?  (2) Participated in any group activities organized by your neighborhood that were aimed at improving or benefitting your neighborhood (e.g. trash clean up, flower/tree planting, building cleanup, neighborhood fundraiser, block party)?  (3) Attended any town/city hall or local government meetings out of concern for an issue in your neighborhood (e.g. crime, availability/quality of local services)?  (4) Attended any organized political event, rally, or protest for an issue that concerns your neighborhood? |

**Exposure Mediator Interaction**

**Table S2. Mediating effects of worrying about food money between neighborhood social capital and tooth decay with interaction between neighborhood social capital and worrying about food money for Oregon Medicaid-enrolled adolescents ages 12 to 18 years who participated in a neighborhood oral health study in 2015-2016 (n = 299)**

| **Neighborhood social capital^1^** | **Proposed mediator** | **Decayed tooth surfaces** | | | | | | |
| --- | --- | --- | --- | --- | --- | --- | --- | --- |
|  |  | **Total effect**  **(95% CI)** | **p** | **NIE^3^**  **(95% CI)** | **p** | **NDE^4^**  **(95% CI)** | **p** | **% Mediated** |
| **Social support** | **Worrying about food money^2^** | 0.04 (-0.29, 0.42) | .85 |  |  |  |  |  |
|  | **Yes** |  |  | -0.02 (-0.13, 0.08) | .67 | 0.06 (-0.26, 0.44) | .73 | 0.9% |
|  | **No** |  |  | -0.02 (-0.18, 0.08) | .65 | 0.06 (-0.25, 0.44) | .76 | 1.8% |
| **Informal social control** | **Worrying about food money** | 0.26 (-0.004, 0.57) | .054 |  |  |  |  |  |
|  | **Yes** |  |  | -0.006 (-0.06, 0.04) | .84 | 0.27 (0.01, 0.60) | .040 | 0.3% |
|  | **No** |  |  | -0.01 (-0.10, 0.05) | .67 | 0.26 (0.002, 0.57) | .048 | 2.1% |

NIE, natural indirect effect; NDE, natural direct effect.

^1^Neighborhood social capital measures are coded so higher values indicate higher social capital.

^2^Worry about food money was dichotomized (never or sometimes vs. often) when modeled as an outcome.

^3^The NIE can be interpreted as the effect of neighborhood social capital on tooth decay that operates through the mediator.

^4^The NDE can be interpreted as the effect of neighborhood social capital on tooth decay if food insecurity did not cause the mediator.

Poisson regression was used to model worrying about food money and tooth decay. All models were adjusted for neighborhood-level and individual-level confounders: neighborhood median income, neighborhood rurality, child age, and child sex. Neighborhood was treated as a random effect to account for clustering. Estimated effects for the mediation analyses are reported on the additive scale (mean differences in the number of untreated decayed tooth surfaces) and estimated at the mean level of confounders, or the most frequent level of categorical confounders.

**DMFS as Outcome**

**Table S3. Mediating effects of worrying about food money between neighborhood social capital and DMFS for Oregon Medicaid-enrolled adolescents ages 12 to 18 years who participated in a neighborhood oral health study in 2015-2016 (n = 299)**

| **Neighborhood social capital^1^** | **Proposed mediator** | **DMFS** | | | | | | |  |
| --- | --- | --- | --- | --- | --- | --- | --- | --- | --- |
|  |  | **Total effect**  **(95% CI)** | **p** | **NIE^3^**  **(95% CI)** | **p** | **NDE^4^**  **(95% CI)** | **P** | **% Mediated** | |
| Social support | Worrying about food money^2^ | 0.14 (-0.85, 1.29) | .82 | -0.06 (-0.29, 0.14) | .50 | 0.19 (-0.75, 1.31) | .73 | 0.5% | |
| Informal social control | Worrying about food money^2^ | 0.38 ( -0.46, 1.32) | .41 | -0.06 (-0.31, 0.15) | .53 | 0.44 (-0.36, 1.36) | .29 | 5.2% | |

DMFS, decayed missing and filled tooth surfaces; NIE, natural indirect effect; NDE, natural direct effect.

^1^Neighborhood social capital measures are coded so higher values indicate higher social capital.

^2^Worry about food money was dichotomized (never or sometimes vs. often) when modeled as an outcome.

^3^The NIE can be interpreted as the effect of neighborhood social capital on tooth decay that operates through the mediator.

^4^The NDE can be interpreted as the effect of neighborhood social capital on tooth decay if food insecurity did not cause the mediator.

Poisson regression was used to model worrying about food money and tooth decay. All models were adjusted for neighborhood-level and individual-level confounders: neighborhood median income, neighborhood rurality, child age, and child sex. Neighborhood was treated as a random effect to account for clustering. Estimated effects for the mediation analyses are reported on the additive scale (mean differences in the number of untreated decayed tooth surfaces) and estimated at the mean level of confounders, or the most frequent level of categorical confounders.
