# Supplementary material for: Associations between neighborhood social capital, oral health risk factors, and tooth decay among Medicaid-enrolled adolescents: A hypothesis-generating preliminary study
Source: PLoS One. 2025 Aug 13;20(8):e0329830. doi: 10.1371/journal.pone.0329830 (PMC12348974; doi:10.1371/journal.pone.0329830)
Supplement: Attachment — Submitted filename: Peer Review Report_PONE_2025.pdf [file pone.0329830.s005.pdf]

Peer Review Report

Manuscript Title: Associations between neighbourhood social capital, oral health risk factors, and tooth decay among Medicaid-enrolled adolescents: a hypothesis-generating preliminary study

Summary

This manuscript explores the association between neighbourhood social capital and untreated tooth decay among Medicaid-enrolled adolescents, with mediation analyses examining how food insecurity and other oral health risk factors might explain this relationship. The revised version addresses key reviewer comments from the initial submission, with expanded explanations, clarified methodology, and improved discussion.

1. Technical Soundness and Statistical Rigor

The study uses appropriate statistical methods, including GLMM and causal mediation analysis based on the potential outcomes' framework. The authors correctly account for clustering and adjust for key confounders. However, a few limitations remain:

i. The sample size (n = 299) limits statistical power, particularly for detecting mediation effects.

ii. The cross-sectional design restricts causal inference, which the authors now appropriately acknowledge.

iii. The neighbourhood-level social capital measures, though theoretically grounded, may be subject to nonresponse bias due to low informant participation (mean response rate: 11%).

Assessment: Technically sound, but interpretation should be appropriately cautious due to data and design constraints.

2. Revisions and responsiveness to reviewer feedback

The authors have been thorough in responding to comments:

i. Clarified the role and relevance of Medicaid in the abstract.

ii. Enhanced the description and justification of the conceptual model and statistical methods.

iii. Added context for key findings (e.g., interpreting a 26% reduction in food insecurity).

iv. Introduced a more holistic view of oral health as part of general health within a social determinant's framework.

v. Added the suggested citation (Silva et al. 2025) and addressed redundancy in the Methods section.

These revisions improve the clarity, relevance, and policy orientation of the manuscript.

3. Remaining issues and suggestions

i. **Generality of the results**: While the study focuses on Medicaid-enrolled adolescents in Oregon, results may not be generalizable to other populations. The authors could briefly emphasize this in the discussion.

ii. **Low power of mediation analysis**: Though acknowledged, it would help if the authors explicitly stated that observed null indirect effects should be interpreted cautiously due to underpowering.

iii. **Figure for GLMM results**: Reviewer 2 suggested visualizing GLMM results. Although authors explain that figures may not be ideal for continuous predictors, a graphical summary (e.g., caterpillar plot of PRs with CIs) could still aid reader interpretation.

The authors have improved the manuscript significantly and are transparent about limitations.
