# Supplementary material for: Associations between neighborhood social capital, oral health risk factors, and tooth decay among Medicaid-enrolled adolescents: A hypothesis-generating preliminary study
Source: PLoS One. 2025 Aug 13;20(8):e0329830. doi: 10.1371/journal.pone.0329830 (PMC12348974; doi:10.1371/journal.pone.0329830)
Supplement: Attachment — Submitted filename: Response to Reviewers.docx [file pone.0329830.s004.docx]

Journal requirements:

When submitting your revision, we need you to address these additional requirements.

1. Please ensure that your manuscript meets PLOS ONE's style requirements, including those for file naming. The PLOS ONE style templates can be found at

[https://journals.plos.org/plosone/s/file?id=wjVg/PLOSOne_formatting_sample_main_body.pdf](https://urldefense.com/v3/__https:/journals.plos.org/plosone/s/file?id=wjVg*PLOSOne_formatting_sample_main_body.pdf__;Lw!!K-Hz7m0Vt54!nkPKwBzzP1EfStIXqlYJCb1j8MIyPKqArqF0e-5DTSHRgO33bNUihPw03-AaEaPi9AcIuvYlRGVR$) and

[https://journals.plos.org/plosone/s/file?id=ba62/PLOSOne_formatting_sample_title_authors_affiliations.pdf](https://urldefense.com/v3/__https:/track.editorialmanager.com/CL0/https:*2F*2Fjournals.plos.org*2Fplosone*2Fs*2Ffile*3Fid=ba62*2FPLOSOne_formatting_sample_title_authors_affiliations.pdf/1/010f01951fbae944-a44f1e6c-0408-40c1-a9bb-002a258a63e2-000000/XKvmCsaX8r2Hl_LxgioBvoxve3Zx7cgihOxXCa1tv5Y=198__;JSUlJSUlJQ!!K-Hz7m0Vt54!nkPKwBzzP1EfStIXqlYJCb1j8MIyPKqArqF0e-5DTSHRgO33bNUihPw03-AaEaPi9AcIum4HZRG1$).

**All PLOS ONE style requirements are met.**

2. Please provide captions for Figure 1 in your manuscript.

**The figure caption was added to the manuscript after the text but before the references.**

Additional Editor Comments:

The manuscript is relevant and brings important discussions to public health. Two reviewers have delivered their assessment, and have suggested some points for improving the manuscript. One main point that should be taken into consideration is the understanding of oral health in the context of comprehensive health and how social capital can be increased or improved.


[Note: HTML markup is below. Please do not edit.]

Reviewers' comments:

Reviewer's Responses to Questions

**Comments to the Author**

1. Is the manuscript technically sound, and do the data support the conclusions?

The manuscript must describe a technically sound piece of scientific research with data that supports the conclusions. Experiments must have been conducted rigorously, with appropriate controls, replication, and sample sizes. The conclusions must be drawn appropriately based on the data presented.

Reviewer #1: Yes

Reviewer #2: Partly

2. Has the statistical analysis been performed appropriately and rigorously?

Reviewer #1: Yes

Reviewer #2: Yes

3. Have the authors made all data underlying the findings in their manuscript fully available? **We can do this – need to restrict dataset to the variables that we analyzed and make sure it’s completely de-identified.**

The [PLOS Data policy](https://urldefense.com/v3/__http:/track.editorialmanager.com/CL0/http:*2F*2Fwww.plosone.org*2Fstatic*2Fpolicies.action*23sharing/1/010f01951fbae944-a44f1e6c-0408-40c1-a9bb-002a258a63e2-000000/gbm4iYPinTwMvOLnaj2IKodVEIZjQ_OFu93U0VuA6iA=198__;JSUlJSU!!K-Hz7m0Vt54!nkPKwBzzP1EfStIXqlYJCb1j8MIyPKqArqF0e-5DTSHRgO33bNUihPw03-AaEaPi9AcIuo-wQOY_$) requires authors to make all data underlying the findings described in their manuscript fully available without restriction, with rare exception (please refer to the Data Availability Statement in the manuscript PDF file). The data should be provided as part of the manuscript or its supporting information, or deposited to a public repository. For example, in addition to summary statistics, the data points behind means, medians and variance measures should be available. If there are restrictions on publicly sharing data—e.g. participant privacy or use of data from a third party—those must be specified.

Reviewer #1: Yes

Reviewer #2: No

4. Is the manuscript presented in an intelligible fashion and written in standard English?

PLOS ONE does not copyedit accepted manuscripts, so the language in submitted articles must be clear, correct, and unambiguous. Any typographical or grammatical errors should be corrected at revision, so please note any specific errors here.

Reviewer #1: Yes

Reviewer #2: Yes

5. Review Comments to the Author

Please use the space provided to explain your answers to the questions above. You may also include additional comments for the author, including concerns about dual publication, research ethics, or publication ethics. (Please upload your review as an attachment if it exceeds 20,000 characters)

**We appreciate the reviewers’ thoughtful comments and feedback on our manuscript. Below we provide responses to queries and suggestions posed by the reviewers. We appreciate the opportunity to improve upon our manuscript.**

Reviewer #1: Review of the manuscript titled "Associations between neighborhood social capital, oral health risk factors, and tooth decay among Medicaid-enrolled adolescents: a hypothesis-generating preliminary study"

The article is well-written and addresses an important topic in public health. It provides a solid theoretical framework and a well-organized structure. A few minor suggestions are offered with the intention of enhancing the text, and I recommend a brief revision to further strengthen the manuscript.

Suggestions:

Introduction
The introduction is well-structured, providing a clear contextual review and a well-defined objective. However, while the study's aim is clear, it could be emphasized more directly. I suggest reformatting the final paragraph to more explicitly highlight the study’s contribution.

**We highlighted the contribution of the study in the final paragraph of the introduction: “The study contributes to efforts examining social capital as a determinant of oral health and aims to identify potential mediators of the relationship.” (lines 98-100)**

Methodology and Results
The methodology is well-described, presented clearly and in detail, and supported by references to studies that utilized similar variables. This allows readers to understand how the study was conducted and the evidence base supporting the methodological choices. However, regarding the results description, my suggestion is that the authors focus more on interpreting the findings, rather than merely replicating the results from the tables. For instance, when presenting the finding "support had a lower prevalence of worrying about food money (prevalence ratio [PR] 0.74; 95% CI 0.56, 0.96; p=.02)," it would be helpful to explain what this really means. This will help contextualize the finding, and it would be even more enriching if the authors could explain, in an accessible manner, what this 26% reduction represents in the context of the study population.

**We added additional text to the results to expand on what a prevalence ratio of 0.74 means: “This means that households in neighborhoods with higher social support reported on average were 26% less likely to experience worry about food money (a form of food insecurity).” (lines 296-298)**

Discussion
The discussion is well-structured and addresses important aspects. However, it is crucial for the authors to consider adding a paragraph that discusses a more integrated perspective, taking into account shared risk factors such as food insecurity, stress, and socio-economic conditions, which affect not only oral health but also other aspects of overall health. Oral health should be approached within a broader context that recognizes the interaction between different social determinants and the need for intersectoral public policies. This point could be highlighted in the "Strengths and Limitations" section or following it, emphasizing that, while social capital plays an important role, it needs to be understood within a broader set of health determinants such as education, income, and access to services. Additionally, how could social capital be integrated into public health strategies? This should also be addressed more thoroughly in the discussion.

**We added a paragraph after strength/limitations that discusses an integrated perspective to promote oral health and potential strategies to address social capital:**

**“Public Health and Policy Implications**

**To fully appreciate the relevance and findings of this work, it is crucial to consider the shared risk factors of oral health and systemic health, including socioeconomic settings that include but are not limited to the experience of financial, housing, and food insecurity and access to education, employment opportunities, social services, and health care. Further, oral health researchers and practitioners must approach prevention and treatment in an integrative manner acknowledging the contextual determinants of health and the interwoven relationship of oral and systemic health. In terms of public health strategies, social capital can be leveraged by modifying social norms for optimal oral health behaviors or via training for organizing and collective action to improve neighborhood conditions.^61^” (lines 463-472).**

**The introduction also describes the role social capital in improving community advocacy: “Similarly, in a neighborhood with high levels of organization participation, community advocacy efforts can secure better resources in the neighborhood like access to healthier food stores, fluoridated tap water, and community clinics that provide comprehensive dental care services. In neighborhoods with high informal social control, adherence to social norms that include healthy behaviors (i.e., healthy eating versus sugary snacks and fast food family; routines like eating meals together every day; or enforcement of oral hygiene behaviors like regular tooth brushing and dental care) may also improve oral health outcomes.” (lines 82-89)**

Reviewer #2: Title: Associations between neighborhood social capital, oral health risk factors, and tooth decay among Medicaid-enrolled adolescents: a hypothesis-generating preliminary study

A relatively recent research topic, the article is relevant when bringing together neighborhood, adolescents and oral health - using mixed model effects. However, it does not bring significant news from a scientific point of view and presents relevant methodological limitations.

Abstract
Considering Medicad as a specific US program, I suggest explaining it in the abstract.

**We added text to the abstract to state that “Medicaid is a public insurance program in the U.S. providing dental insurance to low-income children” (line 9-10).**

Introduction
Page 4 Line 29 – “Atleast half of the U.S. population aged 12-19 years has treated or untreated tooth decay” It is necessary to explain when this data was presented.

**The data presented are from 2018 analyses of nationally representative 2015-2016 data:** “**At least one-half of the U.S. population aged 12-19 years has treated or untreated tooth decay, according to the most recent national estimates from 2015-2016.^5^” (line 31).**

Page 5 Line 61 - If there is a systematic review on social capital and oral health in adolescents, the need for another study is not clear.
I also suggest adding the study by Silva et al. 2025 - da Silva PM, Knorst JK, Ardenghi TM, Tomazoni F. Relationship among different levels of individual and community social capital with oral health related-quality of life among adolescents. J Dent. 2025 Jan;152:105487. doi: 10.1016/j.jdent.2024.105487. Epub 2024 Dec 3. PMID: 39626348.

**The cited systematic review includes 7 studies that examined social capital at a contextual level, of which 4 included objective assessments of oral health. Even after critically reviewing the results of the systematic review, there are a number of questions which warrant further investigation. For example, what is the appropriate level at which to assess social capital (individual vs. contextual)? In addition, how should social capital be measured? Most studies in the review paper used only indicators or proxies that were hypothesized to be related to social capital rather than multiple dimensions of neighborhood social capital developed via theory like in our analysis. Our study also aims to further identify potential neighborhood, household, and individual factors that could mechanistically explain an association between social capital and oral health.**

**We incorporated the Silva 2025 study in the introduction: “…and lack of individual or community social capital was associated with lower oral health related quality of life.^16^” (lines 61-63)**

Methods
The Methods needs to be rewritten. There is a lot of repeated information. Example, Line 130 and Line 137; Line 137 and Line 165.
I suggest following the STROBE ([https://www.strobe-statement.org/](https://urldefense.com/v3/__https:/www.strobe-statement.org/__;!!K-Hz7m0Vt54!nkPKwBzzP1EfStIXqlYJCb1j8MIyPKqArqF0e-5DTSHRgO33bNUihPw03-AaEaPi9AcIuj31i2-R$)) to describe. Information is repeated and inconsistent.

**We removed the sentences from the “adolescent data collection” section that referenced the period of data collection and the specific measures that were collected from adolescents to avoid repeated information.**

Page 7 Line 112 – “Our multilevel study”. Is it a multilevel or multicentric study?

**A multilevel study typically refers to a study in which there are variables assessed at multiple levels and oftentimes when group levels (or units) are at a lower level nested within units at a higher level (Diez Roux 2000). Our study is multilevel because we collected data at multiple levels – individual (oral health), household (routines, food insecurity), and neighborhood (social capital). We added the Diez Roux reference to our manuscript for clarity (line 115).**

Data collection was carried out in 2015 to 2016. Wouldn't the results achieved be outdated today (10 years later)?

**It is possible that certain aspects of the neighborhoods and individuals under study have changed. However, the overall goal of the manuscript was to study how social capital relates to oral health among adolescents and the underlying social, economic, and biologic mechanisms that explain that relationship. These should not have fundamentally changed since the time of data collection.**

The data analysis method is robust, a fact that adds value to the research in question.
Results
There are variables shown in results that are not shown in methods, for example (line 279) " 24% had irregular dental care".

**Notes under Table 1 state that, “To measure whether adolescents had irregular dental care use, we asked caregivers on the caregiver survey, “Does your child currently see a dentist for regular checkups?” A “no” response was considered irregular dental care.” For clarity, we added this to the revised manuscript text (lines 233-236).**

How was family routine assessed and measured? It's not clear.

**The manuscript text states that family routines were assessed using the family routines index (FRI): “Two types of routines were assessed: inconsistent general family routines and inconsistent oral health routines. We measured the former using a modified version of the validated Family Routine Inventory (FRI) which measures cohesion, solidarity, order, and overall satisfaction with family life.^39^ The FRI contains 26 items. Three items were not applicable to adolescents (e.g., “parents read or tell stories to the children almost every day”) and thus removed. Response options were almost never, 1-2 times a week, 3-5 times a week, always/everyday. A composite score was created by summing the responses for the remaining 23 items and was reverse-coded so larger values indicated lower endorsement and adherence to a routine. The range of possible scores was 0 to 69 and missing item-level data were counted as 0.” (lines 188-196).**

I suggest presenting the result of the GLMM model in figure.

**Typically, regression results are reported in a table. If primary predictor variables were categorical, we could use a figure to display the estimated marginal means. However, in our manuscript, the social capital measures are continuous, so this is not an option.**

Considering the magnitude of the research and the high number of variables, it is necessary to review methods and results. Describe in more detail the materials and methods. Focus on the objective.

**We reviewed the methods and results sections carefully editing for clarity, removing repeat information, and ensuring that descriptions were consistent. A few small edits were made to remove extraneous text, describe additional details, and better organize text.**

Discussion
Page 22 Line 340 - “The connection between social support and food insecurity has been previously reported in the literature29-31,51”. Considering this the main result of the study, are there advances in the scientific literature with the results presented?
Avoid repeating results information in the discussion. For example, Line 351-352.

**Our study not only demonstrated the connection between social support and food insecurity, but provided evidence to the hypothesis that experience of household food insecurity could be one mechanism by which a social capital exerts influence on oral health. We removed the line that summarized the finding about food insecurity and tooth decay: “Living in a household that worried about food money often was associated with 2.6 times the decayed tooth surfaces”**

Conclusion
Page 28 Line 460 - “Oral health disparities persist in the U.S. and social determinants like social capital may be an important risk factor to target in future oral health interventions”. Is this conclusion supported by your results?
The conclusion should only respond to the objective.

**We removed the statement about oral health disparities and social determinants.**
