# Supplementary material for: Associations between neighborhood social capital, oral health risk factors, and tooth decay among Medicaid-enrolled adolescents: A hypothesis-generating preliminary study
Source: PLoS One. 2025 Aug 13;20(8):e0329830. doi: 10.1371/journal.pone.0329830 (PMC12348974; doi:10.1371/journal.pone.0329830)
Supplement: Attachment — Submitted filename: Response_to_Reviewers_auresp_2.docx [file pone.0329830.s006.docx]

3. Remaining issues and suggestions

i. Generality of the results: While the study focuses on Medicaid-enrolled adolescents in Oregon, results may not be generalizable to other populations. The authors could briefly emphasize this in the discussion.

**We revised the discussion to acknowledge and comment on the generalizability of the findings: “*However, further research is needed on populations beyond Medicaid-enrolled adolescents to evaluate the generalizability of these findings*.” (lines 358-360).**

ii. Low power of mediation analysis: Though acknowledged, it would help if the authors explicitly stated that observed null indirect effects should be interpreted cautiously due to underpowering.

**We added a statement to the discussion to state that the null indirect effects should be interpreted with caution due to limited power“…*and the null findings should be interpreted cautiously due to our underpowered sample size*” (line 365-366).**

iii. Figure for GLMM results: Reviewer 2 suggested visualizing GLMM results. Although authors explain that figures may not be ideal for continuous predictors, a graphical summary (e.g., caterpillar plot of PRs with CIs) could still aid reader interpretation.

**In table 2, our statistical modeling included log-binomial regression for prevalence ratios (PRs) and linear regression for beta coefficients, resulting in estimates on different scales (multiplicative vs. additive), which complicates graphical summarization. Additionally, Table 3 presents only six PRs, which we believe are sufficiently interpretable without a figure. Thus, we opted not to include a graphical summary, as it may not add clarity in this context.**

The authors have improved the manuscript significantly and are transparent about limitations.

**Thank you for reviewing our manuscript and providing comments to strengthen it.**
